# Global trends in exclusive breastfeeding

**DOI:** 10.1186/1746-4358-7-12

**Published:** 2012-09-28

**Authors:** Xiaodong Cai, Tessa Wardlaw, David W Brown

**Affiliations:** 1 United Nations Population Fund, 605 Third Avenue, New York, NY, 10158, USA; 2 United Nations Children’s Fund, 3 UN Plaza, New York, NY, 10017, USA

**Keywords:** Exclusive breastfeeding, Rate, Trends, Developing countries, Household survey

## Abstract

**Background:**

Infant and young child feeding is critical for child health and survival. Proportion of infants 0–5 months who are fed exclusively with breast milk is a common indicator used for monitoring and evaluating infant and young child feeding in a given country and region. Despite progress made since 1990, a previous review in 2006 of global and regional trends found improvement to be modest. The current study provides an update in global and regional trends in exclusive breastfeeding from 1995 to 2010, taking advantage of the wealth of data from recent household surveys.

**Methods:**

Using the global database of infant and young child feeding maintained by the United Nations Children’s Fund, the authors examined estimates from 440 household surveys in 140 countries over the period between 1995 and 2010 and calculated global and regional averages of the rate of exclusive breastfeeding among infants 0–5 months for the two time points to assess the trends.

**Results:**

Trend data suggest the prevalence of exclusive breastfeeding among infants younger than six months in developing countries increased from 33% in 1995 to 39% in 2010. The prevalence increased in almost all regions in the developing world, with the biggest improvement seen in West and Central Africa.

**Conclusions:**

In spite of the well-recognized importance of exclusive breastfeeding, the practice is not widespread in the developing world and increase on the global level is still very modest with much room for improvement. Child nutrition programmes worldwide continue to require investments and commitments to improve infant feeding practices in order to have maximum impact on children’s lives.

## Background

Infant and young child feeding is critical for child health and survival. Based on well-established evidence, the World Health Organization (WHO) and the United Nations Children’s Fund (UNICEF) recommend that mothers put newborns to the breast within one hour of birth, breastfeed infants exclusively for the first six months and continue to breastfeed for two years and beyond, together with nutritionally adequate, safe, age-appropriate, responsive feeding of solid, semi-solid and soft foods starting in the sixth month [1,2]. There has been growing evidence of the significant impact of early initiation of breastfeeding, preferably within the first hour after birth, on reducing overall neonatal mortality [3-5]. For the first six months of life, breast milk alone is the ideal nourishment, providing all of the nutrients, including vitamins and minerals, an infant needs, meaning that no other liquid or food is needed [1]. In addition, breast milk carries antibodies from the mother that help combat disease, protecting babies from diarrhoea and acute respiratory infections [6]. Breastfeeding also stimulates an infant’s immune system and response to vaccination and, according to some studies, confers cognitive benefits as well [7-10]. Continued breastfeeding beyond six months, accompanied by sufficient quantities of nutritionally adequate, safe and appropriate solid, semi-solid and soft foods, also helps ensure good nutritional status and protects against illnesses. It has been estimated that optimal breastfeeding of children under two years of age has the potential to prevent 1.4 million deaths in children under five in the developing world annually [11].

Unfortunately, early cessation of breastfeeding in favor of commercial breast milk substitutes, introduction of liquids such as water and juices, needless supplementation and poorly timed introduction of solid, semi-solid and soft foods, often of poor quality, is far too common. Although progress has been made since the 1990s, prior reviews [12] of global trends highlight modest improvements in the prevalence of exclusive breastfeeding among children aged less than six months.

In the current study, we aimed at updating the global and regional trends in the prevalence of exclusive breastfeeding among children aged less than six months during the 15-year period from 1995 to 2010.

## Methods

UNICEF maintains a global database of internationally comparable indicators for monitoring infant and young child feeding practices. This database contains data from 440 national household surveys for 140 countries and is updated annually using objective criteria to assess data quality. These data are used to annually report current levels of breastfeeding at global, regional and country levels [13]. Many of the feeding practice indicator data come from UNICEF-supported Multiple Indicator Cluster Survey (MICS) and United States Agency for International Development (USAID) supported Demographic and Health Survey (DHS), which use a standard set of questions on feeding practices during the 24 hours preceding the survey interview, described in detail elsewhere [12]. Country-specific estimates of exclusive breastfeeding rates are calculated according to the standard definition, specified by UNICEF and WHO [14]. Data from nationally representative household surveys other than the MICS and DHS are also included in the UNICEF global database if the data are collected, and indicators computed, using a similar methodology. Detailed information about the MICS and the DHS is available online [15,16]. The current study, a secondary data analysis, was exempt from ethical review as it involved studying existing data that were completely anonymised and delinked from the originating survey participants.

In this review, trends in the prevalence of exclusive breastfeeding among infants younger than six months are reported using data from a subset of countries where at least two comparable data points were available for each country. In total, data were included from 66 countries covering 74% of the developing world population, excluding China for which trend data are currently not available.

Trends at country level were computed by extrapolating existing survey estimates to the baseline year at 1995 and to the follow-up year at 2010 based on the average annual rate of change for each country between available survey data points [12]. Resulting country estimates for 1995 and 2010 were then aggregated to compute regional and global totals using population-weighted averages, where the population weight is the number of births in each country at a given year, obtained from the United Nations Population Division [17], as a proxy for the number of infants 0 to 5 months old. Regional classifications are published by UNICEF in its annual flagship publications [18]. No statistical tests were performed to determine significance of the regional and global trends. It is recognized that uncertainty around the country-specific estimates may be substantial given that the number of infants younger than six months included in the sampling frame of a standard MICS and DHS can be relatively small.

## Results

**Figure 1:**
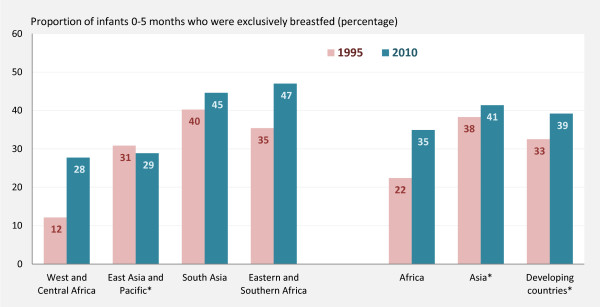
**Trends in exclusive breastfeeding among infants younger than six months.** *Excluding China. Note: Trend analysis based on 66 countries covering 74% of developing world population (excluding China). Trend estimates for Middle East and North Africa and Latin America and Caribbean were not presented due to insufficient data. Source: MICS, DHS and other national household surveys, around 1995 to around 2010, with additional analysis by UNICEF.

Trend data suggest the prevalence of exclusive breastfeeding among infants younger than six months in developing countries increased from 33% in 1995 to 39% in 2010 (Figure 1). The prevalence increased in almost all regions in the developing world, with the biggest improvement seen in West and Central Africa where the prevalence of exclusive breastfeeding more than doubled from 12% in 1995 to 28% in 2010. Eastern and Southern Africa also realized improvements with an increase from 35% in 1995 to 47% in 2010. More modest improvements were observed in South Asia (40% in 1995; 45% in 2010).

Due to lack of trend data from China, there was insufficient data to describe regional patterns for East Asia and Pacific. Excluding China, the prevalence in East Asia and Pacific remained roughly unchanged at around 30% during the past 20 years. Similarly, there was insufficient data for the Middle East and North Africa, Latin America and Caribbean and the Central and Eastern Europe regions (owing largely to a lack of surveys during 1995 and 2010 in a number of populous countries).

**Figure 2:**
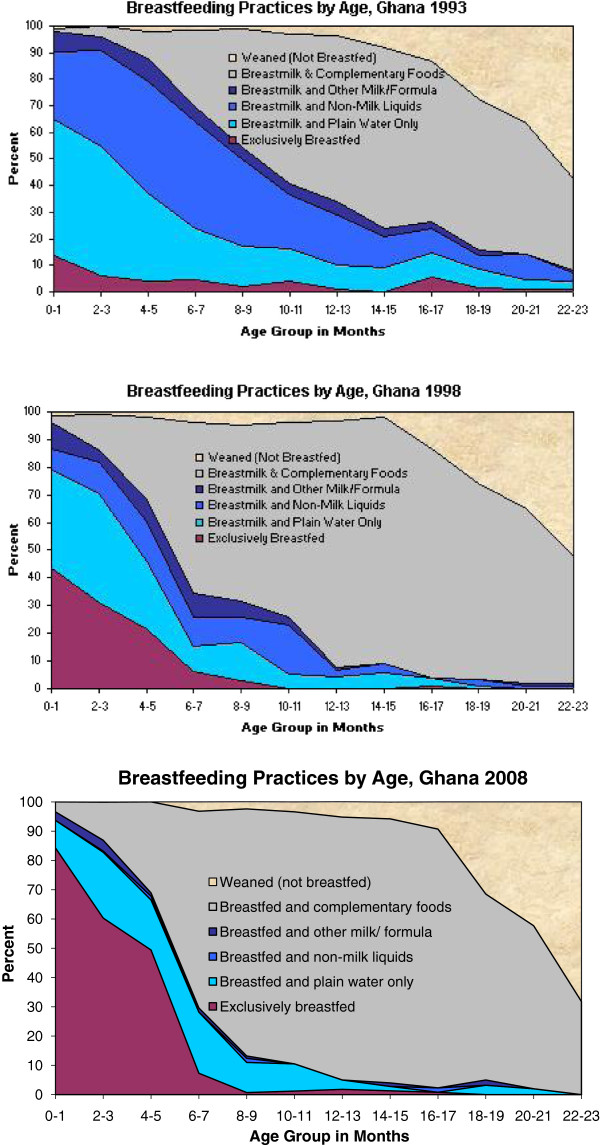
Trends in infant and young child feeding practices, Ghana, 1993 – 2008.

An additional review of infant and young child feeding area graphs of countries with improvements by more than 20 percentage points suggests that the increase in exclusive breastfeeding was due primarily to reductions in suboptimal feeding practices, such as consumption of water, non-milk liquids (e.g. juice) and formula, as shown for Ghana in Figure 2. Information about infant and young child feeding area graphs and qualitative assessment of such graphs is published online [19,20].

## Discussion

Although considerable improvements have been made in some regions, the prevalence of exclusive breastfeeding remains far too low in many areas of the developing world. Our review of data from 66 countries covering 74% of the developing world population – an increase in data coverage from the 38 countries reported previously by Labbok and colleagues [12], observed suboptimal coverage of exclusive breastfeeding, with less than 40% of infants younger than six months of age estimated to be exclusively breastfed in 2010. This is far below the widely accepted “universal coverage” target of 90% coverage [21] and suggests the need for an urgent acceleration of efforts to scale up effective programs in promoting exclusive breastfeeding [22].

While the observed trends lend support to the role of policies and programs focused on breastfeeding – such as the Baby-Friendly Hospital Initiative (BFHI) and the International Code of Marketing of Breastmilk Substitutes [23,24] supported by UNICEF, WHO and other partners, it is, of course, impossible to make causal statements based on these data. Causal inference about the linkage between policy and programme and improvements in exclusive breastfeeding would require additional country-specific qualitative and quantitative analyses, including a review of infant and young child feeding area graphs [19,20] and analysis of disparity and population characteristic data [25,26] that fall beyond the purpose of this brief report.

## Conclusions

In spite of the well-recognized importance of exclusive breastfeeding, the practice is not widespread in the developing world. Others have estimated that suboptimal breastfeeding practices are responsible for more than 1 million child deaths annually and even more striking levels of childhood morbidity [11]. In line with the Global Strategy put forward 20 years ago [2], UNICEF’s strategy and actions in support of infant and young child feeding underline the importance of multi-sectoral approaches to improve health and nutrition and support of proven activities at national, health system and community levels, such as behaviour and social change related to optimal infant feeding practices [27]. If we are clear-sighted in our vision for improving the nutritional status of children worldwide and the importance of the first 1,000 days of life from conception to two years of age [28], then we will acknowledge there is much room for improvement. Child nutrition programmes worldwide continue to require investments and commitments to improve infant feeding practices in order to have maximum impact on children’s lives.

## Competing interests

The authors declare that they have no competing interests.

## Authors’ contributions

XC carried out data analysis and drafted the manuscript. TW conceived of the study and participated in its design and coordination and reviewed the manuscript. DWB made substantial contributions to the interpretation of results and discussions and critically revised the manuscript. All authors read and approved the final manuscript.

## References

[B1] Butte NF Lopez-Alarcon MG Garza C Nutrient adequacy of exclusive breastfeeding for the term infant during the first six months of life 2002 Geneva, Switzerland: World Health Organization

[B2] World Health Organization United Nations Children’s Fund: Global Strategy for Infantand Young Child Feeding 2003 Geneva, Switzerland: World Health Organization

[B3] Edmond KM Zandoh C Quigley MA Amenga-Etego S Owusu-Agyei S Kirkwood BR Delayed breastfeeding initiation increases risk of neonatal mortality Pediatrics 2006 117 e380 e386 10.1542/peds.2005-1496 16510618

[B4] Mullany LC Katz J Li YM Khatry SK Leclerq SC Darmstadt GL Tielsch JM Breast-feeding patterns, time to initiation, and mortality risk among newborns in southern Nepal J Nutr 2008 138 599 603 18287373 10.1093/jn/138.3.599 PMC2366167

[B5] Singh K The effect of colostrum on infant mortality: urban rural differentials Health and Population 1992 15 94 100

[B6] Hanson LA Immunobiology of Human Milk 2004 1 Pharmasoft Publishing, Amarillo, Texas, USA

[B7] Dorea JG Breastfeeding is an essential complement to vaccination Acta Paediatr 2009 98 1244 1250 10.1111/j.1651-2227.2009.01345.x 19594471

[B8] Drane DL Logemann JA A critical evaluation of the evidence on the association between type of infant feeding and cognitive development Paediatr Perinat Epidemiol 2000 14 349 356 10.1046/j.1365-3016.2000.00301.x 11101022

[B9] Mortensen EL Michaelsen KF Sanders SA Reinisch JM The association between duration of breastfeeding and adult intelligence JAMA 2002 287 2365 2371 10.1001/jama.287.18.2365 11988057

[B10] Anderson JW Johnstone BM Remley DT Breast-feeding and cognitive development: a meta-analysis Am J Clin Nutr 1999 70 525 535 10500022 10.1093/ajcn/70.4.525

[B11] Black RE Allen LH Bhutta ZA Caulfield LE de Onis M Ezzati M Mathers C Rivera J Maternal and child undernutrition: global and regional exposures and health consequences Lancet 2008 371 243 260 10.1016/S0140-6736(07)61690-0 18207566

[B12] Labbok MH Wardlaw T Blanc A Clark D Terreri N Trends in exclusive breastfeeding: findings from the 1990s J Hum Lact 2006 22 272 276 10.1177/0890334405279256 16885487

[B13] United Nations Children’s Fund Current status and progress in infant and young child feeding 2012 http://www.childinfo.org/breastfeeding_status.html

[B14] World Health Organization United Nations Children’s Fund: Indicators for assessing infantand young child feedingindicators 2008 Geneva, Switzerland: World Health Organization

[B15] United Nations Children’s Fund Multiple Indicator Cluster Surveys 2011 http://www.childinfo.org/mics.html

[B16] Measure DHS DHS Overview 2011 http://www.measuredhs.com/What-We-Do/Survey-Types/DHS.cfm

[B17] United Nations Population Division World Population Prospects: The 2010 Revision 2011 New York, United States: United Nations Population Division

[B18] United Nations Children’s Fund The State of the World’s Children 2011 New York, United States: United Nations Children’s Fund

[B19] United Nations Children’s Fund Introduction to interpreting area graphs for infant and young child feeding 2010 New York, United States: United Nations Children’s Fund

[B20] United Nations Children’s Fund Infant feeding patterns by country 2012 http://www.childinfo.org/breastfeeding_infantfeeding.html

[B21] Jones G Steketee RW Black RE Bhutta ZA Morris SS How many child deaths can we prevent this year? Lancet 2003 362 65 71 10.1016/S0140-6736(03)13811-1 12853204

[B22] United Nations Children’s Fund, AED Consolidated report of six-country review of breastfeeding programmes 2010 New York, United States: United Nations Children’s Fund

[B23] World Health Organization United Nations Children’s Fund: Baby-Friendly Hospital Initiative 2009 Geneva, Switzerland: World Health Organization

[B24] World Health Organization International code of marketing of breast-milk substitutes 1981 Geneva, Switzerland: World Health Organization

[B25] Grummer-Strawn LM The effect of changes in population characteristics on breastfeeding trends in fifteen developing countries Int J Epidemiol 1996 25 94 102 10.1093/ije/25.1.94 8666510

[B26] Lutter CK Chaparro CM Grummer-Strawn LM Increases in breastfeeding in Latin America and the Caribbean: an analysis of equity Health Policy Plan 2011 26 257 265 10.1093/heapol/czq046 20876642

[B27] United Nations Children’s Fund Infant and young child feeding -- UNICEF response 2012 http://www.unicef.org/nutrition/index_24819.html

[B28] The 1,000 days partnership Why 1,000 days? 2012 http://www.thousanddays.org/about/

